# Supplementary material for: Crystal structures of DNA polymerase I capture novel intermediates in the DNA synthesis pathway
Source: eLife. 2018 Oct 19;7:e40444. doi: 10.7554/eLife.40444 (PMC6231770; doi:10.7554/eLife.40444)
Supplement: Supplementary File 1. — b.Structures of Bst DNAP-I with homologous active sites. [file elife-40444-supp1.docx]

**
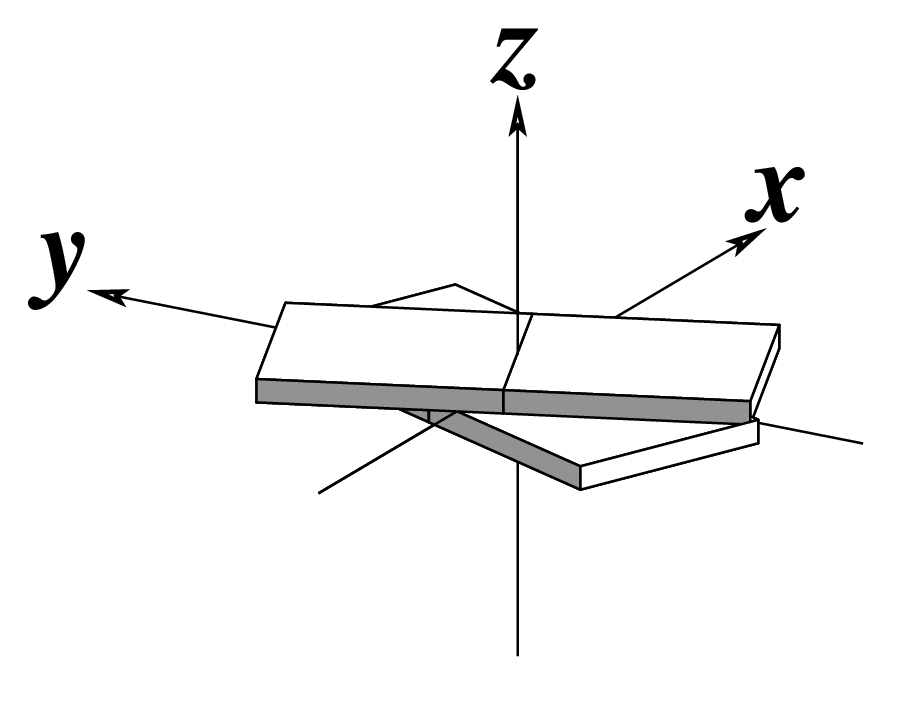

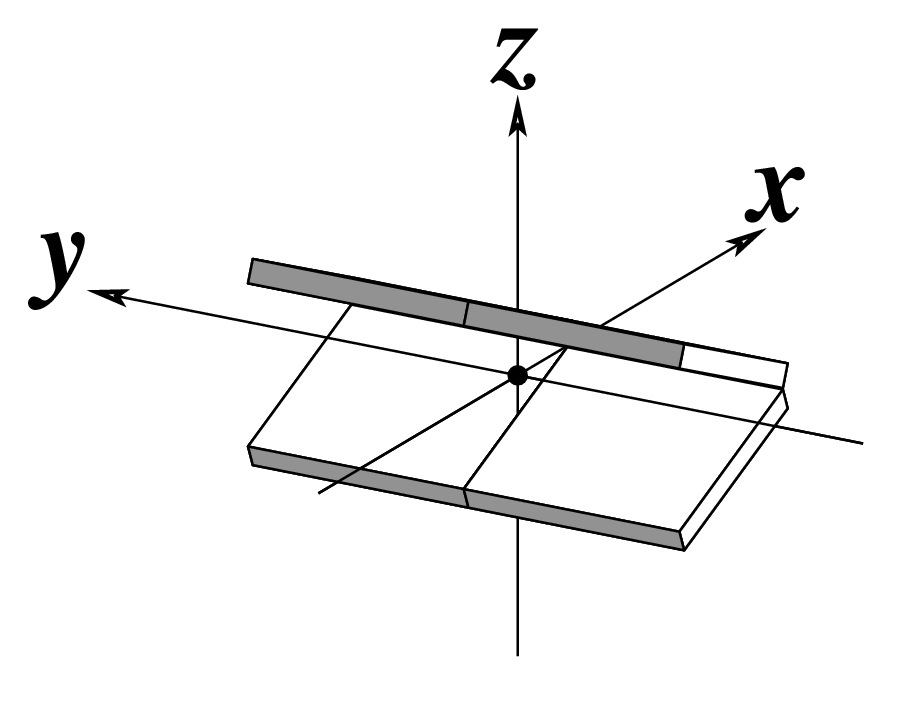

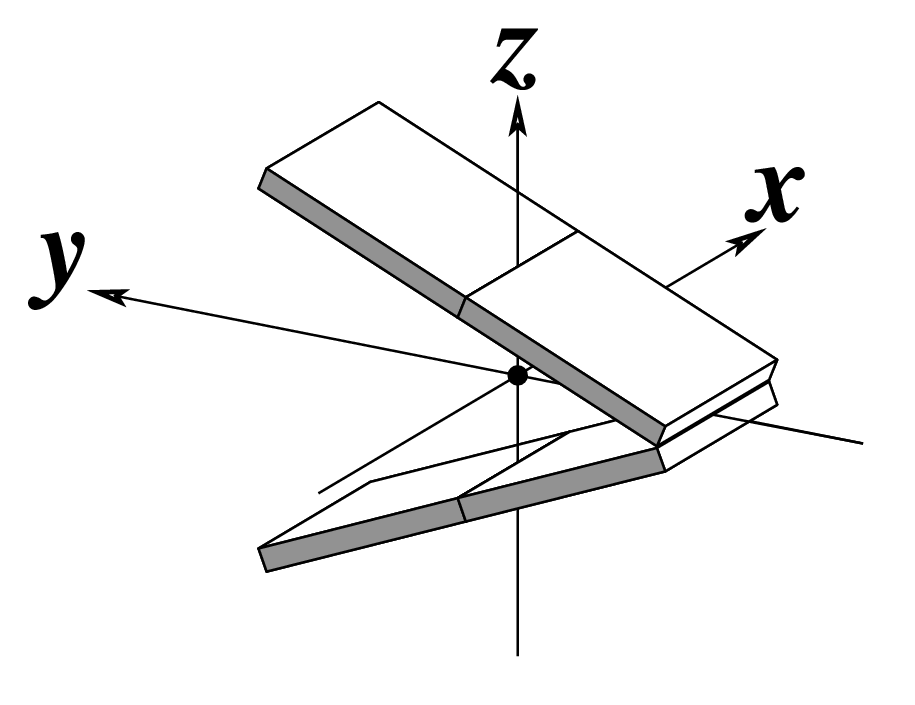

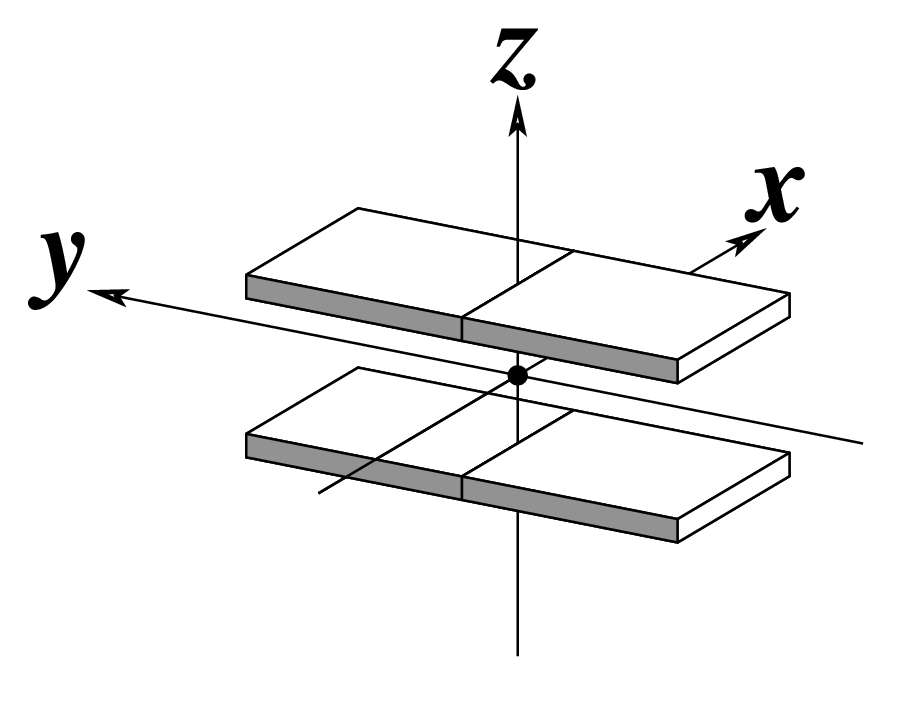

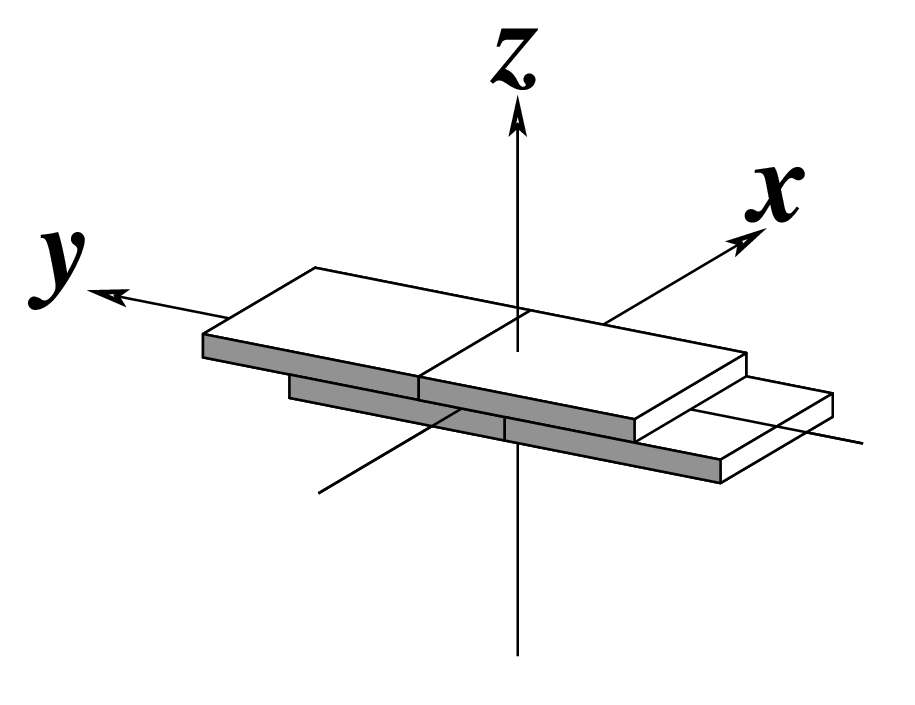

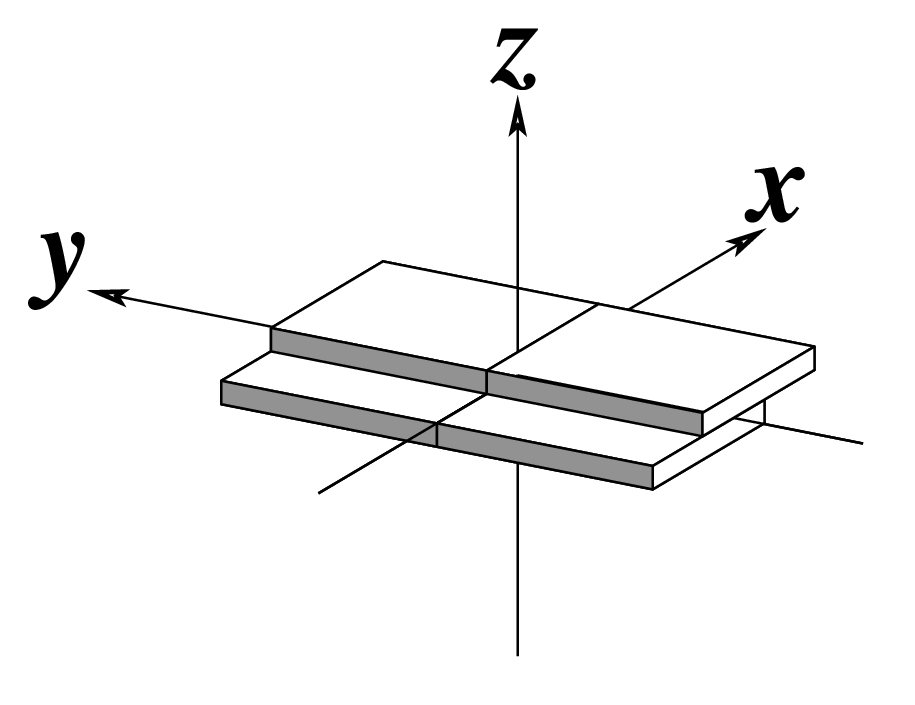
Supplementary File 1a.** Helical statistics for solution and *in crystallo* catalyzed n+1 translocated structures.

Rise

Slide

Tilt

Roll

Twist

Shift

| Primer (DNA) | *In crystallo* (1L3T) | In solution (6DSY) |
| --- | --- | --- |
| **G1** | **C15** | **C15** |
| Shift (Å) | 0.05 | -2.71 |
| Slide (Å) | -0.98 | 0.54 |
| Rise (Å) | 3.05 | 2.94 |
| Tilt (°) | 7.01 | 9.10 |
| Roll (°) | 0.93 | 8.52 |
| Twist (°) | 38.34 | 32.64 |
| **C2** | **G14** | **G14** |
| Shift (Å) | 0.16 | 0.20 |
| Slide (Å) | 1.35 | 0.60 |
| Rise (Å) | 3.30 | 3.63 |
| Tilt (°) | 12.05 | 0.31 |
| Roll (°) | 2.21 | -10.28 |
| Twist (°) | 33.27 | 41.95 |
| **G3** | **C13** | **C13** |
| Shift (Å) | -0.56 | -0.81 |
| Slide (Å) | 0.03 | 0.27 |
| Rise (Å) | 3.26 | 3.32 |
| Tilt (°) | 6.59 | 8.33 |
| Roll (°) | 0.87 | 5.74 |
| Twist (°) | 37.28 | 34.88 |
| **A4** | **T12** | **T12** |
| Shift (Å) | -2.20 | -2.11 |
| Slide (Å) | 0.20 | 0.41 |
| Rise (Å) | 2.84 | 3.19 |
| Tilt (°) | 11.14 | 8.15 |
| Roll (°) | -0.77 | 4.43 |
| Twist (°) | 29.11 | 29.51 |
| **T5** | **A11** | **A11** |
| Shift (Å) | 0.07 | -2.07 |
| Slide (Å) | -1.52 | -1.36 |
| Rise (Å) | 3.69 | 3.20 |
| Tilt (°) | 2.32 | 8.32 |
| Roll (°) | -1.36 | -10.37 |
| Twist (°) | 41.88 | 27.98 |
| **C6** | **G10** | **G10** |
| Shift (Å) | -4.48 | -0.88 |
| Slide (Å) | 1.87 | -0.20 |
| Rise (Å) | 2.42 | 3.16 |
| Tilt (°) | 25.22 | 18.87 |
| Roll (°) | -6.98 | 5.05 |
| Twist (°) | 25.15 | 43.08 |
| **A7** | **T9** | **T9** |
| Shift (Å) | -2.04 | -2.26 |
| Slide (Å) | -0.86 | -0.43 |
| Rise (Å) | 3.08 | 3.36 |
| Tilt (°) | 11.38 | 0.41 |
| Roll (°) | 8.22 | 3.54 |
| Twist (°) | 37.75 | 29.17 |
| **C8** | **G8** | **G8** |
| Shift (Å) | -7.06 | -1.64 |
| Slide (Å) | -0.68 | 0.30 |
| Rise (Å) | 1.91 | 3.71 |
| Tilt (°) | 26.88 | 11.85 |
| Roll (°) | 18.84 | -10.83 |
| Twist (°) | 26.03 | 46.74 |
| **G9** | **C7** | **C7** |
| Shift (Å) | -3.89 | -1.22 |
| Slide (Å) | 1.67 | 0.78 |
| Rise (Å) | 2.67 | 3.82 |
| Tilt (°) | 17.86 | -1.41 |
| Roll (°) | -8.00 | -16.26 |
| Twist (°) | 32.53 | 30.93 |

**Supplementary File 1b.** Structures of Bst DNAP-I with homologous active sites.

| **PDB ID** | **Active Site ‘Distortions’** | **Catalysis Method** |
| --- | --- | --- |
| 1NJW | G:T mismatch at post-insertion site | In crystallo |
| 1NJX | T:G mismatch at post-insertion site | In crystallo |
| 1NK0 | A:G mismatch at post-insertion site | In solution |
| 1NK4 | G:G mismatch at post-insertion site | In solution |
| 1NK6 | C:C mismatch at post-insertion site | In solution |
| 1NK8 | G:T mismatch at post-insertion site | In crystallo |
| 1U47 | C:8oxoG lesion at post-insertion site | In crystallo |
| 1UA1 | C:G-AF (modified G) lesion at post-insertion site | In crystallo |
| 4E0D | E658A mutant | Ternary open |
| 4B9M | T:cFaPydA lesion at post-insertion site | In crystallo and solution |
| 4B9N | T:cFaPydA lesion at n-3 | In crystallo |
| 4B9S | cFaPydG lesion at pre-IS | No catalysis |
| 4B9T | C:cFaPydG lesion at post-insertion site | In crystallo |
| 4B9U | A:cFaPydG mismatch and lesion at post-insertion site | In crystallo |
| 4B9V | A:cFaPydG mismatch and lesion at n-2 | In crystallo |
